# 

**Published:** 1843-02

**Authors:** 


### CONTENTS

OF NO. II.

---

### ORIGINAL COMMUNICATIONS.

### ESSAYS AND CASES.

ART. I.—An Experimental and Critical Inquiry into the Nature and Treatment of wounds of the Intestines. By SAMUEL D. GROSS, M. D., Professor of Surgery in the Louisville Medical Institute. - - - 81

### BIBLIOGRAPHICAL NOTICES.

ART. II.—The Climate of the United States and its Endemic Influences. Based on the records of the Medical Department and Adjutant General's Office, United States Army. By SAMUEL FORRY, M. D. - - 142

### ORIGINAL INTELLIGENCE.

A new Double Salt, the Sulphite of the Protoxide of Platinum and the Sulphite of Soda, discovered and investigated by Messrs. A. Litton and Schnederman. - - - 154
Surgical Diagrams. - - - - - 158
Report of the Charity Hospital, New Orleans. - - 159

